# Genomic landscape of prominent XDR *Acinetobacter* clonal complexes from Dhaka, Bangladesh

**DOI:** 10.1186/s12864-022-08991-x

**Published:** 2022-12-05

**Authors:** Aura Rahman, Ashley Styczynski, Abdul Khaleque, Sakib Abrar Hossain, Abdus Sadique, Arman Hossain, Mukesh Jain, Syeda Naushin Tabassum, Fahad Khan, Mohammad Sami Salman Bhuiyan, Jahidul Alam, Amith Khandakar, Mohammad Kamruzzaman, Chowdhury Rafiqul Ahsan, Saad Bin Abul Kashem, Muhammad E. H. Chowdhury, Maqsud Hossain

**Affiliations:** 1 NSU Genome Research Institute, North South University, Dhaka, Bangladesh; 2 Division of Infectious Diseases and Geographic Medicine, School of Medicine, Stanford University, Palo Alto, California USA; 3 Department of Biochemistry and Microbiology, North South University, Dhaka, Bangladesh; 4 The Hormone Lab & Infertility Centre, Dhaka, Bangladesh; 5 Department of Electrical Engineering, Qatar University, Doha, 2713 Qatar; 6 National Institute of Cancer Research & Hospital, Dhaka, Bangladesh; 7 Department of Microbiology, University of Dhaka, Dhaka, Bangladesh; 8 Department of Computer Sciences, AFG College with the University of Aberdeen, Doha, Qatar

**Keywords:** *Acinetobacter baumannii*, *Acinetobacter nosocomialis*, WGS, Clonal complex, Comparative genomics

## Abstract

**Background:**

*Acinetobacter calcoaceticus-A. baumannii* (ACB) complex pathogens are known for their prevalence in nosocomial infections and extensive antimicrobial resistance (AMR) capabilities. While genomic studies worldwide have elucidated the genetic context of antibiotic resistance in major international clones (ICs) of clinical *Acinetobacter* spp., not much information is available from Bangladesh. In this study, we analysed the AMR profiles of 63 ACB complex strains collected from Dhaka, Bangladesh. Following this, we generated draft genomes of 15 of these strains to understand the prevalence and genomic environments of AMR, virulence and mobilization associated genes in different *Acinetobacter* clones.

**Results:**

Around 84% (*n* = 53) of the strains were extensively drug resistant (XDR) with two showing pan-drug resistance. Draft genomes generated for 15 strains confirmed 14 to be *A. baumannii* while one was *A. nosocomialis.* Most *A. baumannii* genomes fell under three clonal complexes (CCs): the globally dominant CC1 and CC2, and CC10; one strain had a novel sequence type (ST). AMR phenotype-genotype agreement was observed and the genomes contained various beta-lactamase genes including *bla*_OXA-23_ (*n* = 12), *bla*_OXA-66_ (*n* = 6), and *bla*_NDM-1_ (*n* = 3). All genomes displayed roughly similar virulomes, however some virulence genes such as the Acinetobactin *bauA* and the type IV pilus gene *pilA* displayed high genetic variability. CC2 strains carried highest levels of plasmidic gene content and possessed conjugative elements carrying AMR genes, virulence factors and insertion sequences.

**Conclusion:**

This study presents the first comparative genomic analysis of XDR clinical *Acinetobacter* spp. from Bangladesh. It highlights the prevalence of different classes of beta-lactamases, mobilome-derived heterogeneity in genetic architecture and virulence gene variability in prominent *Acinetobacter* clonal complexes in the country. The findings of this study would be valuable in understanding the genomic epidemiology of *A. baumannii* clones and their association with closely related pathogenic species like *A. nosocomialis* in Bangladesh.

**Supplementary Information:**

The online version contains supplementary material available at 10.1186/s12864-022-08991-x.

## Background

*Acinetobacter* is a diverse genus consisting of more than 50 species, the majority of which are harmless environmental bacteria. However, a handful of *Acinetobacter* spp., belonging to the *Acinetobacter calcoaceticus-A. baumannii* (ACB) complex, pose a significant public health burden due to their association with hospital-acquired infections (HAI). Among ACB complex pathogens, *A. baumannii* is frequently encountered in intensive care units (ICUs) of hospitals where it usually infects debilitated patients with impaired host defences and disrupted normal flora [1]. *A. baumannii* most commonly manifests in hospital-acquired pneumonia and bloodstream infections while also contributing to urinary tract infections, meningitis and wound infections. Other members of the ACB complex, i.e., *A. pitti*, *A. calcoaceticus, A. nosocomialis* (formerly known as *A. baumannii* 13TU) and *A. seifertii* are less frequently encountered in the clinical setting but remain central to understanding *Acinetobacter* related disease epidemiology [2, 3].

*A. baumannii* has built a formidable global reputation due its robust antimicrobial resistance (AMR) capabilities against a wide range of antibiotics including carbapenems, making it a priority organism for the World Health Organization (WHO) [4, 5]. Carbapenem resistance in *A. baumannii* is mainly mediated by the carbapenem-hydrolyzing class D beta-lactamases (CHDLs- Ambler class D), which is segregated into several varieties (*bla*_OXA-23-like_, *bla*_OXA-40-like_, bla_OXA-51-like_, *bla*_OXA-58-like_ and *bla*_OXA-143-like_), and less frequently by metallo-beta-lactamases (MBLs- Ambler class B). Since *A. baumannii* is an opportunistic pathogen, it does not usually present traditional pathogenic traits such as toxicity, but instead ensures its persistence in nosocomial settings through immune evasion, biofilm formation and resistance to antimicrobials [6]. *A. baumannii*’s genomic epidemiology is largely defined by a number of dominant international clones (ICs), each consisting of a closely related group of sequence types (STs) [7]. With whole genome sequencing (WGS) becoming an increasingly routine and accessible application in understanding infectious disease epidemiology, many international comparative genomics studies on *A. baumannii* have elucidated the genetic environments of AMR genes (ARGs) and virulence factors in prominent *Acinetobacter* global clones [8–10].

In contrast, there are no genome-scale comparative studies of *A. baumannii* strains in Bangladesh where *Acinetobacter* spp. has been reported as a common causative agent of nosocomial infections [1, 11]. To date, most studies on clinical *Acinetobacter* from Bangladesh have focused only on specific infection types or genes [12–14]. Furthermore, most diagnostic centres do not have access to precise tools for the resolution of different *Acinetobacter* species, which hinders the understanding of the association of various *Acinetobacter* species to nosocomial infections and hospital outbreaks. Due to the lack of genomic data from Bangladesh, there is also a dearth of understanding of the prevalence and resistance patterns of different *Acinetobacter* STs in the country.

In this study, we used WGS data and in vitro phenotypic assays to characterize extensively drug resistant (XDR) clinical *Acinetobacter* spp. belonging to various prominent clonal complexes (CCs) from Dhaka, Bangladesh. We also aimed to analyse the mobile genetic structures and genetic variations within these genomes to understand the genomic architecture of these clones.

## Results

### Antimicrobial susceptibility profile of *Acinetobacter* spp.

**Fig. 1:**
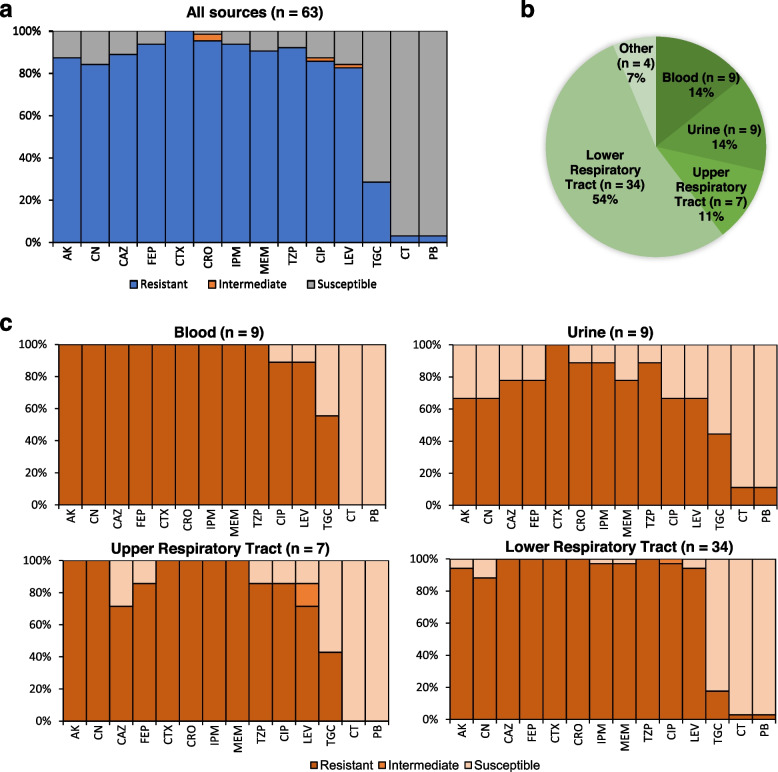
**a.** Antimicrobial susceptibility profile of 63 ACB complex strains against 14 antibiotics. **b.** Source distribution of 63 ACB complex strains **c.** Source-wise antimicrobial susceptibility profiles of the 63 ACB complex strains. AK = Amikacin; CN = Gentamicin; CAZ = Ceftazidime; FEP = Cefepime; CTX = Cefotaxime; CRO = Ceftriaxone; IPM = Imipenem; MEM = Meropenem; TZP = Tazobactam/Piperacillin; CIP = Ciprofloxacin; LEV = Levofloxacin; TGC = Tigecycline; CT = Colistin, PB = Polymyxin B

Sixty-three *Acinetobacter* spp. isolates were collected from diverse clinical sources including tracheal aspirates (*n* = 32), endotracheal tube cultures (*n* = 2), throat swabs (*n* = 7), bile (*n* = 1), wound swab (n = 1), urine (*n* = 9), blood (n = 9) and unspecified clinical sources (n = 2) (Fig. 1). The strains originated from two diagnostic/healthcare centres located in Dhaka, Bangladesh: Dhaka Central International Medical College and Hospital (DCIMCH) and the Hormone Lab and Infertility Center (HLIC). Based on antibiotic susceptibility profiles, all presumptive colonies were multidrug resistant (MDR), with most displaying resistance to third and fourth generation cephalosporins (Additional Table 1). Out of the isolates, 84.1% (*n* = 53) were XDR and showed susceptibility to only polymyxins and tigecyclines. Most strains also displayed resistance against the carbapenems, imipenem (96.8%) and meropenem (93.6%). Susceptibility to polymyxins were high, however, two pan-drug resistant strains were found that exhibited resistance against colistin and polymyxin B (Fig. 1a and Additional Table 1). In addition, 28.6% (*n* = 18) of the isolates showed resistance against tigecycline. Most AMR phenotypes did not appear to be influenced by specimen/source type, although the percentage of tigecycline resistance was higher among urine and blood isolates (~ 53%) in comparison to respiratory isolates (~ 30%) (Fig. 1c). PCR-based amplification of *gyrB* gene fragments inferred all strains to belong to the ACB complex out of which 96.8% (*n* = 61) were detected as *A. baumannii*.

### Sequencing statistics of 15 *Acinetobacter* genomes

A total of 15 strains were sequenced out of which 14 were confirmed as *A. baumannii* using the PCR amplification of *gyrB* gene, while one was a non-*baumannii* bacterium belonging to the ACB complex. Moreover, 10 strains were sequenced from DCIMCH and five from HLIC, while phenotypically 13 were XDR and two were MDR.

BLAST of 16S ribosomal gene fragments and subsequent average nucleotide identity (ANI) analysis confirmed 14 strains to be *A. baumannii* while the one ACB complex strain was discovered to be *A. nosocomialis.* Pan-core analysis of the 14 *A. baumannii* strains showed the core genome to contain 3909 genes and the pangenome to contain 8198 genes. Inclusion of the *A. nosocomialis* strain reduced the core genome size to 3847 genes and raised the pangenome size to 9531 genes. Assembly of the sequenced reads resulted in contig numbers varying from 75 to 202 and N50 values ranging from 55,790–141,320 bp (Table 1). The genome sizes of the 15 strains were ~ 3.6–4.0 Mb and the GC content ranged from 38.66–39.12%, values that are typical for *Acinetobacter* genomes. The *A. nosocomialis* strain had the lowest GC% (38.66%) out of all the strains while its genome size (3,939,631 bp) fell somewhere in the middle of the range. While one pan-drug resistant isolate had been sequenced along with the 15 strains of this study, the assembly revealed contaminations from a co-infection of *Klebsiella* spp., and therefore it was eliminated from the analysis.

**Table 1:** Assembly, annotation statistics and in silico MLST and serotyping of 15 ACB strains

Clonal Cluster	MLST (Pasteur)	Strain	Contigs	Total length (bp)	GC%	N50 (bp)	Genetic Features	Species	KL	OCL
CC1	ST1	NGCE922	75	3,988,373	39.08	134,180	3999	*Acinetobacter baumannii*	17	1
		NGCE923	80	3,989,129	39.08	114,427	4039		17	1
CC2	ST2	NGCE921	117	3,922,910	38.9	141,320	4010		12	1
		NGCE928	105	3,971,924	38.84	110,112	4070		3	1
		NGCE1004	76	3,941,505	38.83	140,356	3907		2	1
		NGCE1005	80	3,941,955	38.83	135,425	3905		2	1
		NGCE1008	84	3,940,652	38.83	122,061	3917		2	1
		NGCE1011	86	3,941,223	38.83	122,061	3914		2	1
CC10	ST10	NGCE927	202	4,077,429	38.89	61,251	4212		9	2
	ST23	NGCE924	133	3,787,997	38.85	59,480	3898		8	2
	ST575	NGCE925	138	3,902,546	38.9	71,877	3932		13	2
		NGCE926	131	3,899,992	38.9	71,877	3928		13	2
CC33	459	CRA-AC-04	136	3,724,429	39	55,790	3778		27	2
–	Novel	CRA-AC-05	115	3,607,524	39.12	69,480	3571		23	6
–	ST768	NGCE1007	100	3,936,364	38.65	139,355	3872	*Acinetobacter nosocomialis*	29	4

### *Acinetobacter* genomes belonged to prominent clonal clusters in Asia

Strains belonging to both *A. baumannii* and *A. nosocomialis* species were classified using Pasteur’s MLST scheme- the gold standard method for sequence typing both *baumannii* and non-*baumannii* isolates [7]. The results showed six strains belonging to ST2, the central ST of clonal complex 2 (CC2) that corresponds to international clone IC2, as per PubMLST’s BURST analysis (Additional Fig. 1a). Two strains were ST1, belonging to CC1 or IC1. The dataset of this study had a total of four strains of CC10: one belonging to the ancestral ST10, and others belonging to ST23 and ST575, both of which are single locus variants (SLVs) of the former. The remaining two *A. baumannii* genomes of the study were classified as the following STs: ST459 which is an SLV of CC33 and a novel ST, respectively. The *A. nosocomialis* strain was of ST768, which is a central ST of a prominent clone for this species (Additional Fig. 1b).

In silico serological typing was also performed on the strains by determining the gene loci of the capsular polysaccharide (KL) and lipo-oligosaccharide (OCL). All ST1 and ST2 strains possessed the OCL locus type 1 (OCL1) while all four CC10 strains possessed OCL2 (Table 1). Much more diversity was observed in KL locus types with 10 different predicted KL types being distributed in the 15 isolates.

### Phylogenetic analysis of 15 *Acinetobacter* genomes

**Fig. 2:**
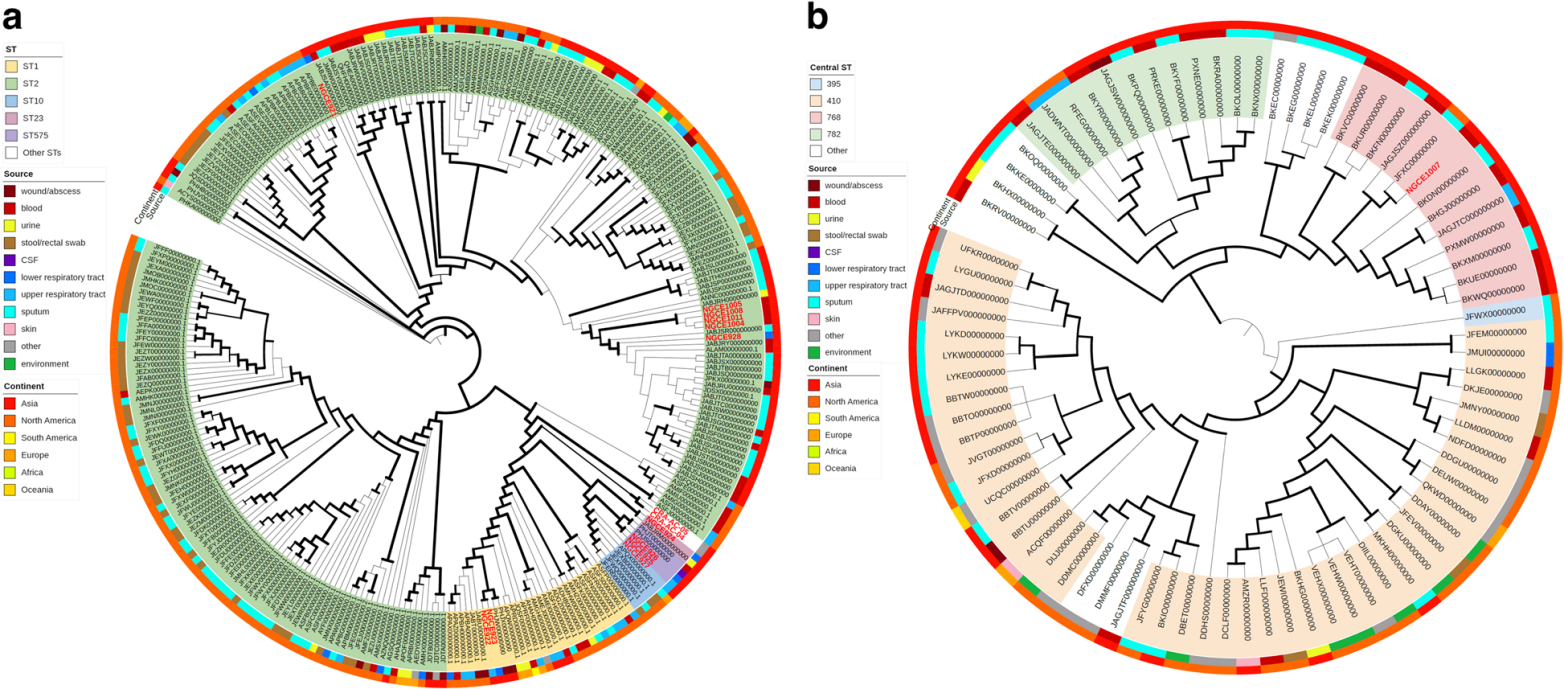
Core-genome maximum likelihood tree of **a)** 14 sequenced *A. baumannii* strains **b)** the *A. nosocomialis* strain NGCE1007 along with other reference strains for each species obtained from NCBI. Core genome alignment was generated with Roary while the ML tree was generated using IQTree with 1000 bootstrap resampling. The trees were rooted at midpoint, visualized and annotated using iTOL. The branch thickness of the trees directly correlates to bootstrap value

For a thorough analysis of phylogenetic and clustering patterns, all publicly available *A. baumannii* strains belonging to any of the STs encountered in this study were included in the dataset (Additional Table 2). Since a large proportion of publicly available *A. baumannii* genomes were ST2, only a few randomly selected strains of that ST belonging to different sources were included. The core-genome SNP-based maximum likelihood tree generated from a total of ~ 300 *A. baumannii* strains showed the grouping of strains belongning to the same CC (Fig. 2a). However, no clear topological distinction between strains originating from different sources was observed.

**Table 2:** Nodes bearing replication initiation protein genes in 15 *Acinetobacter* genomes

Clonal Cluster	MLST (Pasteur)	Strain ID	Node No.	Length (bp)	Conjugative system	AMR genes
CC1	ST1	NGCE922	42	8808	Absent	None
		NGCE923	47	7630	Absent	None
CC2	ST2	NGCE921	23	49,378	Absent	None
			44	8808	Absent	None
		NGCE928	24	65,089	*traG, traH, traN, trbC, traU, traW, traB, traL*	*aph-(3′)-*VIa
			27	49,330	Absent	*armA, msr(E), mph(E)*
			51	12,598	Absent	None
			57	8808	Absent	None
		NGCE1004	24	49,371	Absent	*armA, msr(E), mph(E)*
			33	35,050	*traG, traH, traN, trbC, traU, traW, traB, traL*	None
			50	8808	Absent	None
		NGCE1005	24	49,371	Absent	*armA, msr(E), mph(E)*
			32	35,050	*traG, traH, traN, trbC, traU, traW, traB, traL*	None
			52	8808	Absent	None
		NGCE1008	24	49,371	Absent	*armA, msr(E), mph(E)*
			34	35,050	*traG, traH, traN, trbC, traU, traW, traB, traL*	None
			53	8808	Absent	None
		NGCE1011	24	49,371	Absent	*armA, msr(E), mph(E)*
			33	35,050	*traG, traH, traN, trbC, traU, traW, traB, traL*	None
			56	8808	Absent	None
CC10	ST10	NGCE927	88	9635	Absent	None
			96	6856	Absent	None
			101	6119	Absent	None
			115	3010	Absent	None
	ST23	NGCE924	66	14,956	Absent	None
			67	14,928	Absent	None
			104	1939	Absent	None
	ST575	NGCE925	57	16,753	Absent	*msr(E), mph(E), tet(39)*
			65	11,914	Absent	None
			92	4303	Absent	None
			110	1118	Absent	None
		NGCE926	56	17,671	Absent	*msr(E), mph(E), tet(39)*
			119	523	Absent	None
			129	354	Absent	None
CC33	ST459	CRA-AC-04	77	6899	Absent	None
–	Novel ST	CRA-AC-05	48	23,082	Absent	None
CC768	ST768	NGCE1007	40	16,019	Absent	None
			56	5140	Absent	None

While the goeBURST analysis of all existing *A. baumannii* STs could not connect the novel ST CRA-AC-05 to any clonal cluster network, the core-genome SNP tree placed the former next to CC33 member CRA-AC-04. Cluster analysis based on the accessory genome profiles of 70 selected ACB complex strains mostly concurred with the grouping pattern of the core-genome based phylogenetic tree (Additional Fig. 2). Within the main cluster, two distinct clades were observed, one containing CC10 and CC33 strains and the other containing the CC1 and CC2 subclades. CRA-AC-05 once again clustered next to CC33 strain CRA-AC-04, indicating high similarity in accessory gene content between them. The isolation sources of the strains, as colour-coded in Additional Fig. 2, also showed an intermixing of strains isolated from various anatomical sites.

BURST analysis of *A. nosocomialis* revealed ST768 to be a central ST and therefore an ancestral ST to its clone (Additional Fig. 1b). While the core-genome SNP tree of *A. nosocomialis* strains also did not depict any grouping pattern dependent on source of isolation, strains belonging to clonal clusters CC768 and CC782, the former of which contained NGCE1007, appeared to be predominant in Asia and less frequent elsewhere (Fig. 2b).

### *Acinetobacter* ARG profiles correlate with ST and phenotype

**Fig. 3:**
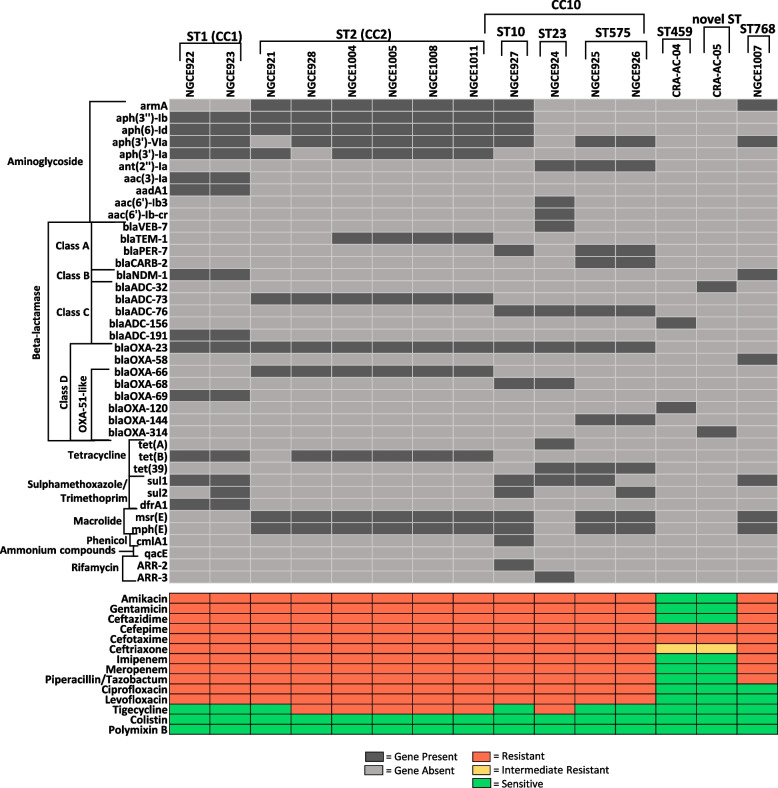
Antimicrobial susceptibility and resistome profiles of 15 sequenced *Acinetobacter* strains. The bipartite heatmap depicts the distribution of antimicrobial resistance genes at the top, and the phenotypic susceptibility status of the 15 sequenced strains against 14 tested antibiotics at the bottom. AMR genes were initially identified using ResFinder database (coverage > 60%; identity > 90%). The specific gene numbers for beta-lactamases were identified using the BLDB BLAST [43]

All 14 *A. baumannii* genomes possessed different varieties of the intrinsic class C ARG *bla*_ADC_, a gene missing from the *A. nosocomialis* strain, NGCE1007 (Fig. 3). Penicillin-hydrolyzing class A beta-lactamases (*bla*_VEB-7_, *bla*_TEM-1_, *bla*_PER-7_ and *bla*_CARB-2_) were limited to CC2 and CC10 strains while the class B MBL *bla*_NDM-1_ was only detected in CC1 strains and the *A. nosocomialis* strain, NGCE1007. All 15 genomes carried various CHDLs: while the XDR CC1, CC2 and CC10 *A. baumannii* genomes contained *bla*_OXA-23_*,* the MDR *A. nosocomialis* strain NGCE1007 was the sole carrier of the *bla*_OXA-58_ type. All *A. baumannii* strains, including carbapenem-susceptible CRA-AC-04 and CRA-AC-05, contained various types of *bla*_OXA-51-like_ beta-lactamase genes. An inter-ST difference in ARG distribution was also observed. The ST1 strains were the sole carriers of *bla*_OXA-69_, sulfamethoxazole/trimethoprim resistance conferring gene *dfrA1* and aminoglycoside resistance genes *aac* [3]*-Ia* and *aadA1*. Several other ARGs that were unique to a particular ST in the dataset include: *bla*_OXA-66_ for ST2 strains, *bla*_OXA-120_ for ST459 strain CRA-AC-04, *bla*_CARB-2_ and *bla*_OXA-144_ for the two ST575 strains, and *bla*_OXA-314_ for the novel ST CRA-AC-05.

Moreover, the ARG profiles corroborated the phenotypic resistance characteristics of the strains (Fig. 3). For CRA-AC-04 and CRA-AC-05, the absence of any aminoglycoside resistance conferring genes was reflected in their susceptible AMR phenotype against the tested antibiotics of this class. These two strains also displayed susceptibility to carbapenems, thus agreeing with their AMR gene profile which did not contain *bla*_OXA-23_. In contrast to the other strains, these two strains along with *A. nosocomialis* strain NGCE1007 also displayed susceptibility to fluoroquinolones, however no differentiating ARG was observed in the other resistant genomes. Ten of the genomes carried various types of *tet* genes, however only NGCE924 possessed *tetA* which has been shown to reduce tigecycline susceptibility in *A. baumannii* [15]*.* None of the analysed genomes carried *tet(X)*, a recently characterised plasmid mediated tigecycline resistance gene family that has been reported in various *Enterobacteriaceae* [16].

### *Acinetobacter* genomes possess a rich virulome

**Fig. 4:**
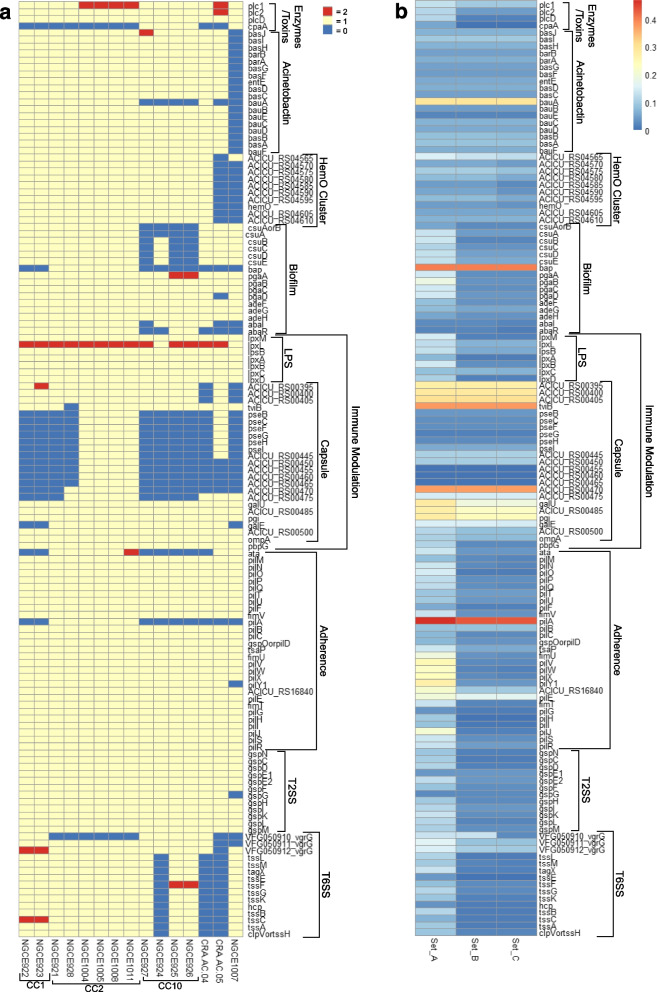
**a)** Virulome profiles of 15 *Acinetobacter* genomes. Virulence gene distribution was determined in by BLAST against 135 *A. baumannii* virulence genes in the VFDB database. Only those genes with the e-value below 1e^−5^, coverage > 90% and identity > 60% were considered. The colours depicted in the heatmap indicate the number of hits for each gene as explained in the legend. **b)** Genetic variance in virulence genes of *Acinetobacter* spp. strains calculated over three sets. Set A contains genes associated with all 15 *Acinetobacter* genomes as well as the VFDB reference gene. Set B contains all genes from Set A except for those associated with *A. nosocomialis* genome NGCE1007. Set C contains only those genes associated with the 14 *A. baumannii* genomes, i.e., sans the VFDB reference gene. The genetic variance was calculated with DnaSP in terms of the ratio SNP/bp where bp stood for the length of the aligned gene. The SNP/bp ratio is represented with a colour scale as shown in the legend

The genomes of the 15 sequenced strains were checked for the presence of 135 *A. baumannii* virulence genes as obtained from the Virulence Factor Database (VFDB) (Fig. 4). Overall, the 15 genomes possessed a rich assortment of virulence genes with all of them containing 46.67% (*n* = 63) of the virulence genes in the dataset. Out of all the virulence gene groups analysed, the Acinetobactin operon (*bauFDCEBA*, *basABCDEFG,* and *barAB*), which is necessary for activating *A. baumannii*’s iron chelating pathway, was the only gene cluster that was exclusively absent in the *A. nosocomialis* genome.

Bacterial adherence and biofilm production in *Acinetobacter* is mediated by several gene groups, such as the resistance-nodulation-cell division (RND) family efflux pump genes *adeFGH*, the Csu pili system genes (*csuABCDE*), the polysaccharide matrix producing *pgaABCD* locus, the type IV pilus (TFP) *pil* genes, the quorum sensing genes *abaI/abaR* and the biofilm associated protein gene *bap* [17–19]. In the phenotypic biofilm formation test, all strains produced mild to moderate biofilms with Specific Biofilm Formation (SBF) value between 0.11–0.89 (Additional Fig. 3). Nearly all of the genes in the *pga* and *adeG* clusters were present in all 15 *Acinetobacter* genomes while the *csu* operon was found missing from most of the strains belonging to CC10. The *bap* gene was found to be exclusive to ST2 strains. Moreover, based on SBF values and virulence gene distribution pattern, no clear correlation could be established between the phenotype and genotype of biofilm formation.

The capsular polysaccharide is a key feature of *A. baumannii,* that aids in its survival in different environments and protects the bacterium against host immune system action such as the serum complement [20]. Among the genes involved with the production of capsular polysaccharide, the enzymatic machinery of *pse* and associated genes which metabolizes UDP-linked sugars were found to be missing from all strains apart from the four ST2 strains from HLIC that had been isolated from blood. Other genes involved in serum survival such as the penicillin-binding protein (*pbpG)* [21], outer membrane protein (*ompA*) were ubiquitously found in the genomes.

Other notable absences of gene groups included the heme utilization HemO gene cluster for *A. nosocomialis* strain NGCE1007 and the novel *A. baumannii* ST CRA-AC-05 and the type VI secretion system (T6SS) genes for NGCE924, CRA-AC-04 and CRA-AC-05.

### Certain virulence genes display increased genetic variance

The relatively high frequency of virulence factors in the core genome of our dataset prompted us to analyse the level of genetic variance within these genes. This was estimated by calculating their SNP/bp ratios over three iterations to adjust for any bias introduced due to inclusion of the *A. nosocomialis* strain and VFDB reference genomes in the dataset. All 15 *Acinetobacter* genomes along with the VFDB reference was included in Set A, following where the *A. nosocomialis* strain NGCE1007 and the VFDB reference were iteratively eliminated from the dataset to produce Sets B and C.

The results revealed increased genetic variances within otherwise conserved operons of *Acinetobacter* spp. and *A. baumannii.* The Acinetobactin *bauA* for example, is a gene belonging to a highly conserved operon of *A. baumannii* and shows high variance with an SNP/bp ratio of 0.294 for Set C. As mentioned before, the TFP gene cluster formed part of *Acinetobacter* core genome. While other genes of this operon were shown to be genetically conserved, *pilA* showed high levels of divergence with SNP/bp ratios ranging from 0.432 to 0.468 across the three tested sets. In case of *pilA,* the increased SNP/bp values resulted from a highly variable tail-end region of the gene which has been well documented in previous literature [22]. The UDP-*N*-acetylglucosamine 6-dehydrogenase gene *tviB* also showed elevated SNP/bp ratio at ~ 0.353 while the *bap* gene wasfound to be highly variant among the ST2 strains (SNP/bp ratio ~ 0.398).

Overall, the analysis also revealed that while most *A. baumannii* virulence operons were present in the *A. nosocomialis* strains, they exhibited genetic divergence from their corresponding *A. baumannii* operons causing SNP/bp ratio to rise above 0.1 in most cases.

### *Acinetobacter* STs display varying levels of similarity to reference plasmids

**Fig. 5:**
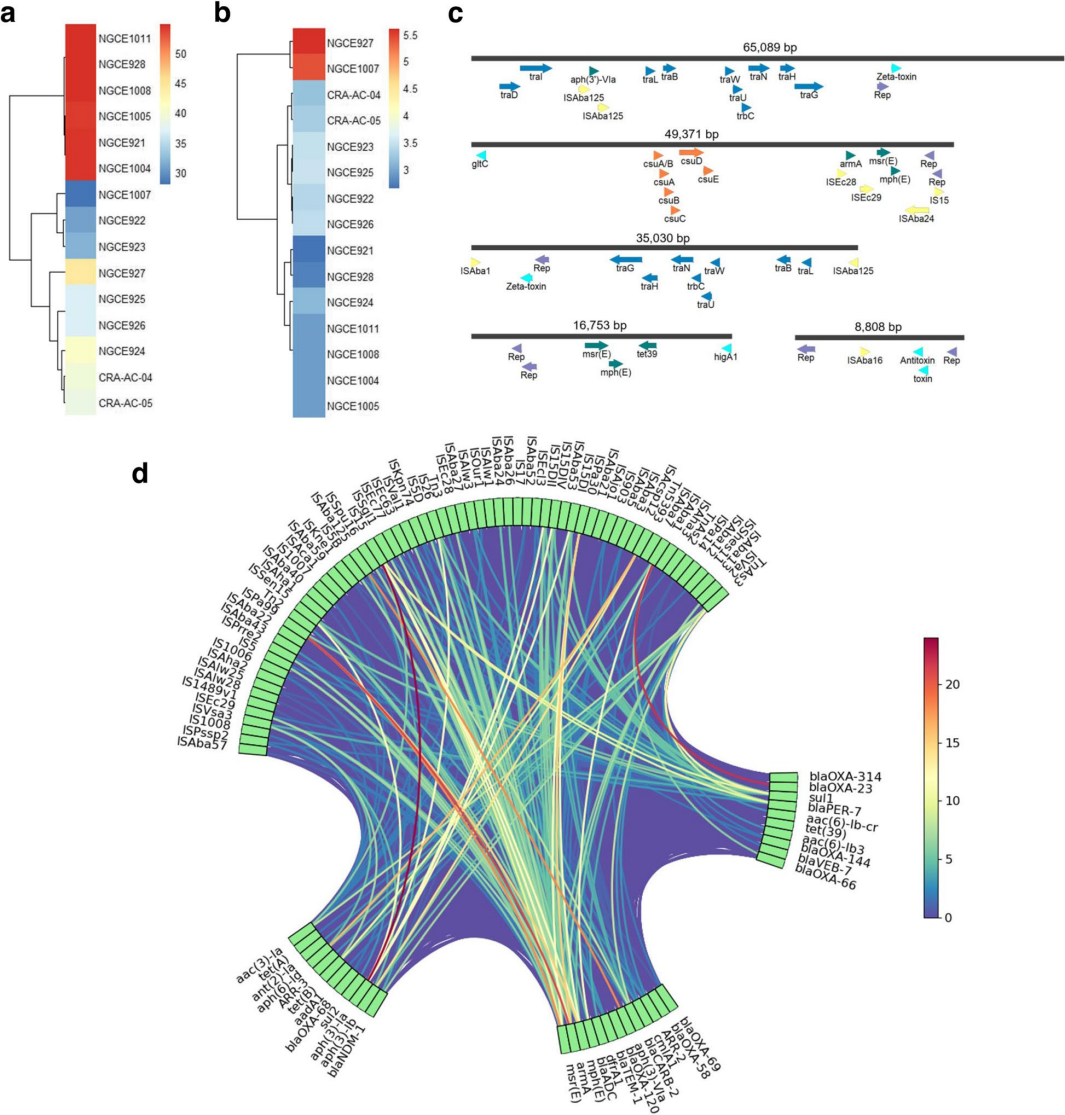
**a)** Level of shared gene content of 15 *Acinetobacter* strains when compared against 578 *A. baumannii* reference plasmids. **b)** Level of shared gene content of 15 *Acinetobacter* strains when compared against 27 *A. nosocomialis* reference plasmids. The numbers on the colour scale represent percentage of the genome that matched against the databases. **c)** Schematic diagrams showing the arrangements of nodes associated with conjugative systems and replicase genes found in common among several *Acinetobacter* genomes. **d)** Relational diagram showing association between insertion sequences (ISs) and various AMR genes. The colour of the links joining AMR genes to ISs represent the number of times the connection appeared among the 15 *Acinetobacter* genomes analysed. The diagram was generated using CIRCOS

BLAST search was performed of the 15 genomes against a reference database of 578 publicly available *A. baumannii* plasmids to estimate the length of the genomes that matched against known plasmid sequences. The fraction of matched genome length was treated as a measure of the genome’s receptivity to plasmidic DNA. The analysis revealed a correlation between ST and the level of shared gene content: among the 15 strains, the five ST2 strains had the highest amount of BLAST similarities in plasmidic gene content, accounting for 2.13–2.18 Mb or ~ 54% of their genomes (Fig. 5a). Another prevalent clone, ST1 on the other hand, had the lowest level of shared gene content, with both NGCE922 and NGCE923 possessing only ~ 31% of genetic similarity with other *A. baumannii* plasmids. *A. nosocomialis* NGCE1007 was most conservative to shared gene content and had only ~ 27% of its genome similar to *A. baumannii* plasmids.

The 15 strains were also matched against a database of 27 *A. nosocomialis*’s plasmids. However, in this case the shared gene content was much lower in comparison to *A. baumannii* plasmid database with *A. nosocomialis* strain NGCE1007 itself showing only ~ 5.59% similarity (Fig. 5b). *A. baumannii* NGCE927 (ST10), however, showed a comparable level of shared gene content to that of the *A. nosocomialis* strain (~ 5.41%). Among the remaining *A. baumannii* strains, the ST2 strains had the lowest level of shared gene content with *A. nosocomialis* plasmids (~ 2.84%), while the rest had slightly higher levels of shared gene content of ~ 3.31%.

### Mobilome architecture of different *Acinetobacter* STs

Limitations of short reads obtained from Illumina sequencing meant that complete plasmid assemblies could not be obtained from the sequencing data. Hence, we opted for an annotation-based approach for the identification of putative integrative and conjugative elements (ICEs) and integrative and mobilizing elements (IMEs) within our genomes. For this, the assembled contigs or nodes of the genomes were first searched for replication initiation proteins. The nodes carrying these proteins were then further analysed to identify genetic factors that are commonly enriched in ICEs/IMEs including conjugation systems, mobilization proteins, and toxin-antitoxin systems. These nodes were also annotated for the presence of ARGs, virulence factors and insertion sequences (ISs).

Among the 15 genomes analysed, all CC2 strains barring NGCE921 carried the F-like conjugation system consisting of *traG, traH, traN, trbC, traU, traW, traB, traL* along with replication initiation proteins (Table 2). The four ST2 strains from HLIC contained a 35,030 bp node carrying the conjugation machinery, and it mapped against a 65,089 bp plasmid node obtained from the ST2 strain NGCE928 from DCIMCH. The 65,089 bp NGCE928 plasmidic node also contained the IS*Aba125*-associated ARG *aph (3′)-*VIa. These CC2 genomes also carried a ~ 49,371 bp node containing two replication initiation proteins as well as the *csu* virulence operon and the IS*Ec29*-IS*Ec28*-IS*Aba24* associated *armA*-*msr(E)*-*mph(E)* ARGs (Fig. 5c). All ST1 (CC1) and ST2 (CC2) strains contained a ~ 8088 bp node while the two ST575 (CC33) contained a ~ 17,000 bp node, both carrying two replicase proteins (Fig. 5c). Analysis via ICEfinder [23], also revealed the CC1 genomes and all CC10 genomes barring NGCE924 to carry putative IMEs consisting of genes coding for mobilization proteins MobA/MobL and integrases. However, no ARGs or virulence factors were found associated with them.

Identification of ISs within genomes, using ISFinder revealed the 15 strains to contain a myriad of ISs (Additional Fig. 4), many of which were found to be linked with ARGs (Fig. 5d). The IS*Aba1* insertion was found flanking *bla*_OXA-23_ for all genomes carrying the gene. On the other hand, for the ST1 strains and *A. nosocomialis* NGCE1007, the *bla*_NDM-1_ gene was carried in a 6388–8076 bp node. The *bla*_NDM-1_ gene was preceded by IS*Aba125* upstream and followed by the TnAs3 transposase downstream. It was also observed that the TnAs3 family transposase played a role in the genetic arrangements of the class A family beta-lactamases *bla*_PER-7_ and *bla*_CARB-2_, the aminoglycoside resistance conferring gene *armA* and the sulbactam resistance conferring gene *sul1* in the CC10 strains, thus contributing to diversity in genetic structures within this resistance island.

## Discussion

Carbapenem resistant *A. baumannii* (CRAB) have become globally rampant in hospital ICUs, challenging empirical treatment conventions and necessitating the use of toxic, last-line therapies such as colistin [24]. The strains of this study displayed high rates of resistance against carbapenems (> 90%), and most other drugs. The frequency of susceptibility to colistin was high, suggesting it can be an effective treatment option ad interim. However, the occurrence of colistin-resistant strains in our samples adds to the growing body of reports of pan-drug resistant *A. baumannii* in the subcontinent [25, 26]. Rising rates of tigecycline resistance among CRAB in recent years is another growing area of concern and our study concurs with recent reports demonstrating the growing frequency of tigecycline resistant CRAB around the world [27, 28]. Altogether the AMR profiles of strains reinstated the escalating prevalence of extensive drug resistance in *Acinetobacter* spp. in Bangladesh, thus necessitating reforms in healthcare delivery, including antibiotic stewardship and enhanced infection control.

All XDR *A. baumannii* genomes analysed in this study fell into CC1, CC2 or CC10, all three of which are prominent *A. baumannii* clones circulating in Asia (Additional Fig. 1a). In addition, this study also reports two MDR strains CRA-AC-04 and CRA-AC-05, which shared greater genetic proximity with CC10 strains, as per both core-genome phylogeny and accessory genome based hierarchical clustering pattern. A number of recent genomic and molecular typing studies originating from China and Southeast Asia have reported on the overwhelming dominance of ST2/CC2 strains in their *A. baumannii* population, while CC1 was found to be completely absent [29, 30]. On the contrary, several Indian studies have reported both CC1 and CC2 strains, albeit the latter was more frequent [31, 32]. While CC2 is ubiquitously predominant across the world, MLST data shows CC1 distribution to be more concentrated in North and South American nations. Our findings, taken together with other studies from neighbouring regions highlight the relatively greater abundance of CC1 in the Indian subcontinent in comparison to Southeast Asian countries. This, taken together with the discovery of several *A. baumannii* genomes belonging to another prevalent Asian clone CC10 and a prevalent *A. nosocomialis* ST (ST768) within our small pool of isolates, can be considered evidence towards the diversity of *Acinetobacter* strains within the country.

*Acinetobacter* spp.’s XDR phenotype is attributed to its genome harboring a wide range of beta-lactamase genes. CHDLs are thought to be the primary genetic determinants of carbapenem resistance. All of our *A. baumannii* strains that carried *bla*_OXA-23_ were phenotypically resistant to the carbapenems imipenem and meropenem whereas strains CRA-AC-04 and CRA-AC-05 lacking them were susceptible to these antibiotics. *A. nosocomialis* lacked *bla*_OXA-23_ but carried *bla*_OXA-58_ suggesting the latter gene might be a key genetic factor behind carbapenem resistance in the species. The class B1 MBL gene *bla*_NDM-1_, another contributor to carbapenem resistance, is particularly prevalent in South Asia and neighbouring regions. Among our strains, *bla*_NDM-1_ was found in both the ST1 strains and the *A. nosocomialis* isolate, but in none of the ST2 strains. Despite lower abundance of *A. baumannii* IC1 and *A. nosocomialis* in the clinical setting, these findings highlight their importance in *Acinetobacter* AMR epidemiology.

While various studies have implicated the roles of *tet(X)* family genes and *tetA* in tigecycline resistance, they were either absent or rare in the genomes analysed in this study. Other reports have identified the overexpression of RND efflux pumps and increased levels of mutations in associated genes as the predominant mechanism of tigecycline resistance in CRAB [28, 33]. Through genetic variation analysis we were able to detect high levels of mutations in *tviB*, supporting a recent report which also links it to reduced tigecycline susceptibility [28].

Virulence mechanisms such as biofilm formation are also strongly linked to the AMR phenotype of *Acinetobacter* spp., and often dictate their success as a nosocomial pathogen in hospital ICUs and NICUs. Although the sample size was too small to draw significant conclusions regarding ST-, source-, and location-specific differences in expressed virulence phenotypes, the strains of this study displayed moderate levels of biofilm formation that is comparable to other published works [34, 35]. Most genomic studies on *A. baumannii* report the ubiquity of virulence factors in its genome, irrespective of infection type and source. In this study too, most genomes were found to be highly enriched with virulence genes. However, the enhanced genetic variability within certain genes of otherwise conserved operons indicates a more complex mechanism of virulence as opposed to a direct causal relationship between gene presence/absence and virulence potential. Further studies on these genetic variabilities could elucidate whether they influence expressional changes in gene clusters involved in *Acinetobacter*’s virulence pathways.

Mobile genetic elements (MGEs) such as plasmids, ICEs, IMEs and ISs partake in horizontal gene transfer and are thus the regulators of the dissemination of virulence and resistance islands among *Acinetobacter* strains. *Acinetobacter*’s plasmidic structures and lineages have been difficult to comprehend due their diversity in terms of composition and size [36]. CC2/IC2 strains possessed highest plasticity to *A. baumannii* plasmidic contents while CC1/IC1 had the lowest, thus showing differing levels of genetic content received from *A. baumannii* plasmids in these two international clones. Moreover, the ST10 strain NGCE927 also revealed similarity to *A. nosocomialis* plasmid databases that was at a comparable level to that of the *A. nosocomialis* strain of this study. Out of the 15 studied genomes we also observed five out of six CC2 genomes to carry a conjugation machinery that was missing in other strains. The study also highlighted the role of ISs in altering the spatial arrangements of ARGs as seen with the transposon TnAs3 among CC10 isolates. Taken together, these insights further support the dynamicity of *Acinetobacter*’s mobilomic structure both between and within CCs.

While we managed to contribute on the genomic structures of Bangladeshi *Acinetobacter* STs belonging to prominent clones, the study is limited by its small sample size. Overall, there is also the need to study probable associations between genetic variances and observed virulence through more in-depth phenotypic analyses and knockout assays. Furthermore, in our study we were only able to analyse putative ICEs/IMEs and draw associations between ISs and ARGs. With different sequencing techniques becoming accessible across the world, hybrid assemblies using short and long reads could be implemented to capture the genetic organization of larger MGEs such as plasmids and accurately resolve the precise genetic environments of important ARGs such as carbapenem resistance genes of the *bla*_OXA_ family.

## Conclusion

In this first-ever comparative genomics study on clinical *Acinetobacter* isolates from Bangladesh, we shed light on the presence of prominent clonal complexes of XDR *Acinetobacter* spp. in the country and reveal genomic insights on their ARGs and virulence factors. In addition, this study also highlights the role of ICEs and ISs in the dissemination of ARGs and virulence factors in *Acinetobacter* spp. The findings of this study are an important standpoint in understanding the epidemiology of *Acinetobacter* infections and their genomic characteristics pertaining to virulence, AMR and MGE activity in the subcontinent. Future genomic studies with a larger sample size and more in-depth analysis of phenotypes will be key in understanding the pathogenesis and resistance mechanisms of clinical *Acinetobacter* spp.

## Methods

### Sample collection, antibiotic susceptibility testing and PCR

Fifty-eight clinical isolates of *Acinetobacter* spp. were collected from inpatients admitted into the ICU, NICU, high dependency units and general medical wards of DCIMCH, Dhaka, Bangladesh, as well as from outpatients visiting the clinic for diagnostic purposes. In addition, five other ACB strains originating from blood samples were acquired from HLIC, an independent diagnostic laboratory in Dhaka. The isolates were collected from various clinical sources as listed in Additional Table 1. All samples were cultured on MacConkey agar (Oxoid, USA), following which biochemical tests were carried out for presumptive identification of *Acinetobacter* spp. The samples were then grown in T1N1 soft agar and transferred to North South University Genome Research Institute (NGRI), Dhaka, Bangladesh for further molecular and genomic characterization.

For all antibiotics except for the polymyxins and tigecycline, the CLSI’s disc diffusion breakpoints for *Acinetobacter* spp. were used [37]. Broth microdilution was carried out for polymyxins and tigecycline using the recommended guidelines by CLSI and EUCAST respectively. To interpret the results for polymyxins, CLSI’s recommended breakpoints for *Acinetobacter* spp. were used (S < 2, and R > 4). In case of tigecycline, since no breakpoints are available for *Acinetobacter* spp., the EUCAST breakpoints for *Enterobacterales* were used (S < 0.5, R > 0.5) [38].

For further molecular confirmation for the ACB complex, DNA was extracted from the cultures of all 63 strains using QIAamp® DNA Mini Kit (Qiagen, Germany) and a conventional PCR assay was performed involving the *gyrB* gene fragments to identify ACB complex strains [39].

### Sequencing, assembly and annotation

A subset of strains was selected for WGS, covering the two centres and varying time-points, sources and AMR phenotypes. Barcoded libraries were prepared using the Illumina DNA Prep workflow and Nextera DNA CD indexes. Paired end sequencing of 2 × 151 cycles was performed on Illumina MiSeq at NGRI. The raw reads were assembled using SPAdes version 3.15.1 [40], with a coverage cut-off of 20x and contigs shorter than 300 bp filtered out. The assembled genomes were annotated using Prokka [41]. The isolates were genotyped in silico using the Pasteur scheme of *Acinetobacter* spp. MLST [42]. The genomic resistance profiles of the strains were uncovered using the ResFinder tool (identity > 90%, coverage > 60%). The genes identified as beta-lactamases by ResFinder were further analysed by performing BLASTx on them against the Beta-Lactamase DataBase (BLDB) in order to correctly identify their gene numbers [43, 44]. VFDB was used to identify virulence genes within the genomes (e-value cut-off 1e^− 5^, identity > 60%, coverage > 90%) [45], and *Acinetobacter* KL and OCL loci were identified using Kaptive [46]. Genetic variations within selected virulence genes were analysed using DnaSP following alignment of genes with MAFFT [47, 48]. Putative ICEs and IMEs were predicted using a combination of the online tool ICEfinder [23], and BLAST. Insertion sequences (ISs) and transposons were identified using ISFinder with e-value cut-off of 1e^− 5^ [49].

### Pan-core, clonal complex, and phylogenetic analysis

Auxilliary information of all publicly avaible *A. baumannii* and *A. nosomialis* strains were collected from PATRIC (up until December 2nd, 2021, [50]. Based on their Pasteur MLST allelic profile, a clonal complex network was built using goeBURST [51].

Pan-core genome analysis of strains was carried out using Roary [52], and a cluster dendrogram based on the presence-absence of accessory genes was generated using the Pvclust package of R which used bootstrap resampling (*n* = 1000) to generate *p-*values [53]. Separate phylogenetic trees were generated for *A. baumannii* and *A. nosocomialis* using the strains sequenced in this study along with representative strains obtained from NCBI (Additional Tables 2 and 3). For this, quick core genome alignments were obtained from the PRANK and MAFFT programs of Roary following which single nucleotide polymorphism (SNP) sites were obtained from the core genome alignment using SNP-sites [54]. Maximum likelihood trees were then built using the ultrafast bootstrapping option of IQTree with 1000 replicates [55]. The resulting trees were visualized and edited with iTOL [56].

### Plasmid analysis

Assembled whole genomes of the strains were also checked for shared gene content with other available *A. baumannii* and *A. nosocomialis* plasmids by performing a BLAST search using an e-value threshold of 10^− 20^ against a database of 578 *A. baumannii* plasmids and 27 *A. nosocomialis* plasmids downloaded from NCBI (Additional Table 4).

### Biofilm formation assay

Biofilm formation assay was performed using a slightly altered version of the previously described protocol [57]. Briefly 2 μL of overnight cultures grown in Luria-Bertani (LB) broth were inoculated in 198 μL of fresh LB in sterile microtiter plates with four replicates per strain. 200 μL LB without any inoculum was used as a blank. The plates were incubated overnight at 37 °C following which the optical density at 600 nm (OD_600nm_) was measured (GloMax Explorer, Promega). The cultures were then discarded and the wells were rinsed twice with 1X phosphate buffered saline (pH 7.4). The plates were air dried and then stained for 15 minutes in 250 μL of 0.1% crystal violet (w/v). The stained biofilm cells attached to the well walls were then released in 200 μL of 33% glacial acetic acid and the plates were read at 560 nm. Due to no universally recognized system existing for evaluation of *Acinetobacter* biofilm formation level, for this study we opted to measure biofilm forming propensity using specific biofilm formation (SBF) value that was described in a previous study [58]. Here, SBF = (AB – CW)/G, where AB is OD_560nm_ of stained cells, CW is OD_560nm_ of control wells, and G is bacterial cell growth calculated using the formula G = OD_600nm(24 hr)_ – OD_600nm(0 hr)_. Strains with SBF value below 0.5 was categorized as mild biofilm formers, those with SBF 0.5 or greater, but less than 1 were categorized as moderate biofilm formers and those with SBF value of 1 or greater were labelled as strong biofilm formers. The entire experiment was repeated twice.

## Supplementary Information

**Additional file 1.** Figure S1-S4

**Additional file 2.** Table S1. Antimicrobial susceptibility of 63 clinical Acinetobacter spp. strains isolated from Dhaka, Bangladesh.

**Additional file 3 **Table S2. Global *Acinetobacter baumannii* strains used for pholygenetic analysis.

**Additional file 4 **Table S3. Global *Acinetobacter nosocomialis* strains used for pholygenetic analysis.

**Additional file 5 **Table S4. NCBI accessions of plasmid sequences of *A. baumannii* and *A. nosocomialis*.

## Data Availability

The genomic sequence data generated in this study have been submitted to NCBI’s GenBank repository (https://www.ncbi.nlm.nih.gov/genbank/) under the accession numbers JAKRBR000000000 to JAKRCF0000000000, as indicated in Additional Table 5.

## Abbreviations

ACB: *Acinetobacter calcoaceticus-A. baumannii*
HAI: Hospital-acquired infection
ICU: Intensive care unit
AMR: Antimicrobial resistance
CHDL: Carbapenem-hydrolyzing class D beta-lactamase
MBL: Metallo-beta-lactamase
IC: International clone
ST: Sequence type
WGS: Whole genome sequencing
ARG: AMR gene
XDR: Extensively drug resistant
CC: Clonal complex
DCIMCH: Dhaka Central International Medical College and Hospital
HLIC: Hormone Lab and Infertility Center
MDR: Multidrug resistance
ANI: Average nucleotide identity
SLV: Single locus variants
KL: Capsular polysaccharide
OCL: Lipo-oligosaccharide
VFDB: Virulence Factor Database
TFP: Type IV pilus
SBF: Specific Biofilm Formation
ICE: Integrative and conjugative element
IME: Integrative and mobilizing element
IS: Insertion sequence
MGE: Mobile genetic element
SNP: Single nucleotide polymorphism
