# Supplementary material for: Genomic landscape of prominent XDR Acinetobacter clonal complexes from Dhaka, Bangladesh
Source: BMC Genomics. 2022 Dec 5;23:802. doi: 10.1186/s12864-022-08991-x (PMC9721023; doi:10.1186/s12864-022-08991-x)
Supplement: Supplementary file 1 — Additional file 1. Figure S1-S4 [file 12864_2022_8991_MOESM1_ESM.pdf]

a)

b)

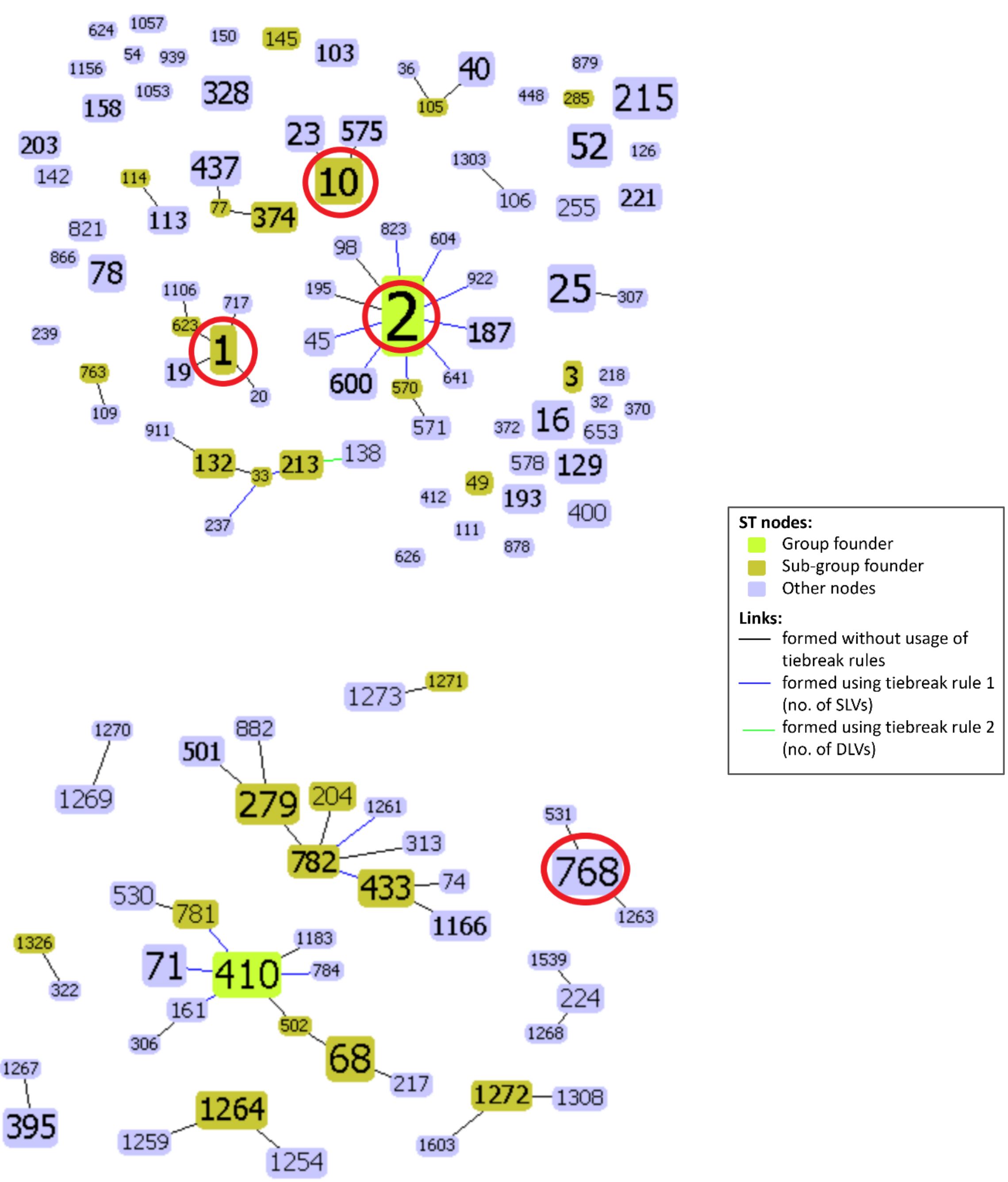

Additional Fig. 1. a) goeBURST snapshot of *Acinetobacter baumannii* genomes available from all Asian countries. **b)** goeBURST snapshot of all *A. nosocomialis* genomes available from around the world. The red circles in both figures highlight the central ST of the clonal complexes that were encountered in this study (CC1, CC2, CC10 for *A. baumannii* and ST768 for *A. nosocomialis*). The size of the nodes in each part of the figure is proportional to its relative abundance in comparison to other strains in the dataset.

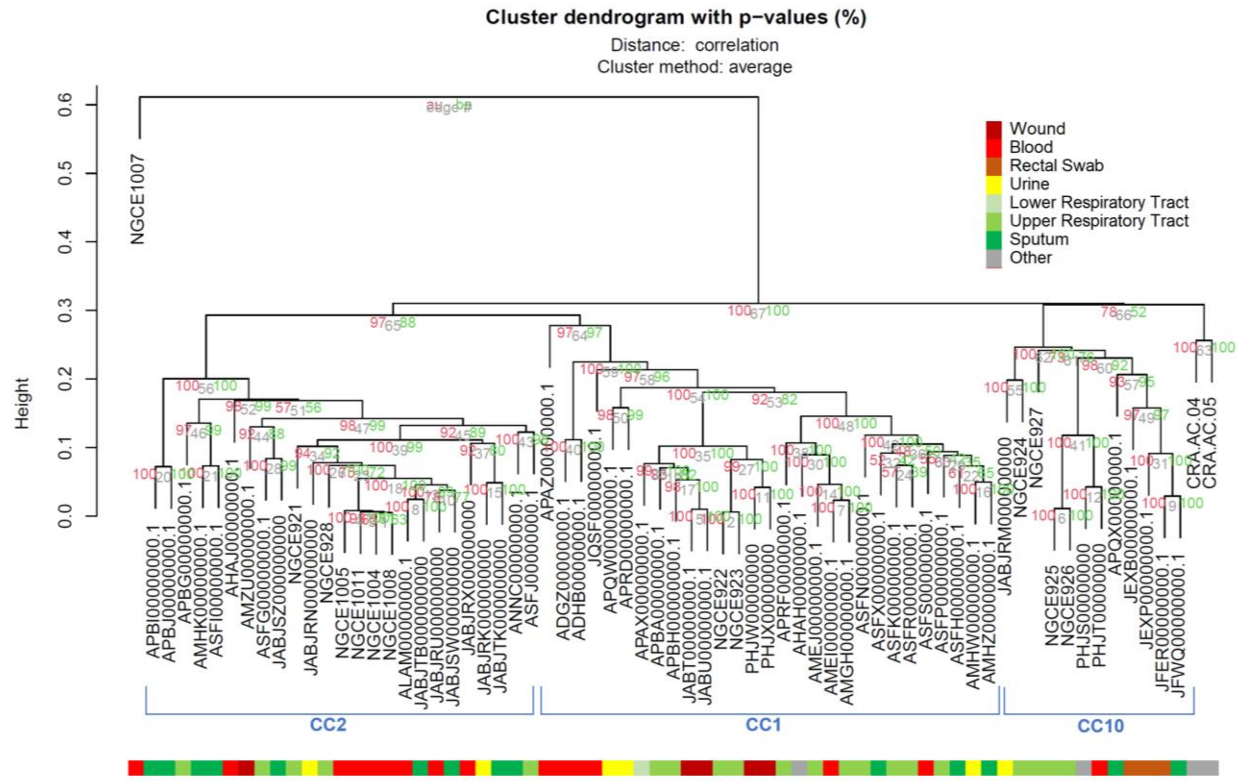

Additional Fig. 2. Cluster dendrogram of 70 ACB isolates based on their accessory gene profile. Isolation source of strains, indicated by colors, show interspersing of strains from different anatomical sites. The dendrogram was generated using the Pvclust package of R. It calculates the bootstrap probability (BP) value, denoted in green, using ordinary bootstrap resampling. The approximately unbiased (AU) *p*-value, denoted in red, is computed using the multiscale bootstrap resampling method.

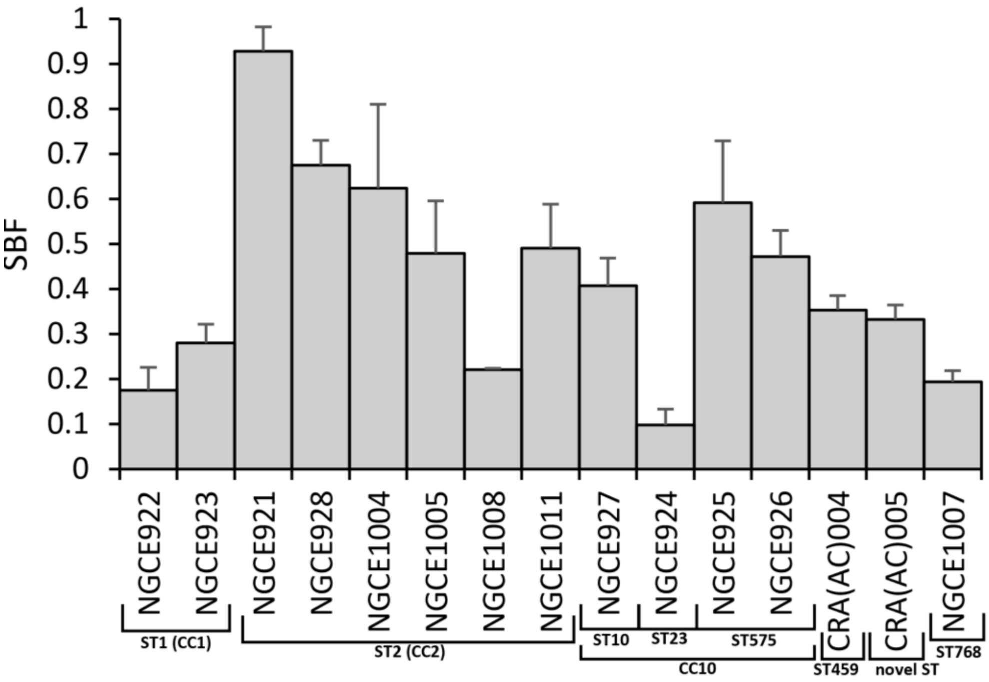

Additional Fig. 3. Specific Biofilm Formation (SBF) values of 15 *Acinetobacter* strains. The error bars represent standard error.

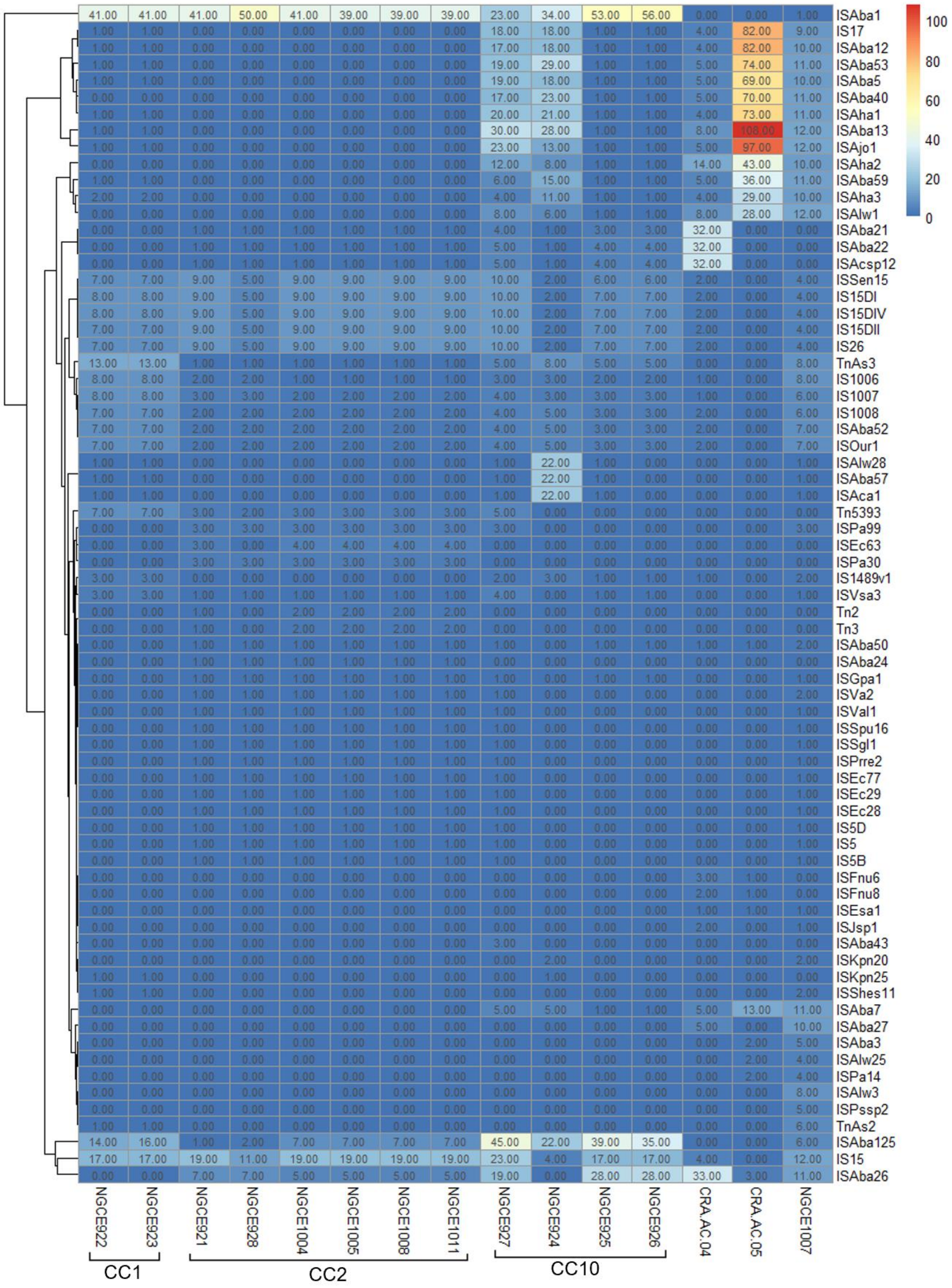

Additional Fig. 4. Distribution of ISs found in association with AMR genes in 15 *Acinetobacter* genomes
